# *In Vitro* Evaluation of Antimicrobial Peptides from the Black Soldier Fly (*Hermetia Illucens*) against a Selection of Human Pathogens

**DOI:** 10.1128/spectrum.01664-21

**Published:** 2022-01-05

**Authors:** Laurence Van Moll, Jeroen De Smet, Anne Paas, Dorothee Tegtmeier, Andreas Vilcinskas, Paul Cos, Leen Van Campenhout

**Affiliations:** a Laboratory for Microbiology, Parasitology and Hygiene (LMPH), Faculty of Pharmaceutical, Biomedical and Veterinary Sciences, University of Antwerp, Antwerp, Belgium; b Department of Microbial and Molecular Systems (M^2^S), Research Group for Insect Production and Processing, KU Leuven, Geel, Belgium; c Leuven Food Science and Nutrition Research Centre (LFoRCe), KU Leuven, Leuven, Belgium; d Fraunhofer Institute for Molecular Biology and Applied Ecology, Branch for Bioresources, Gießen, Germany; e Institute for Insect Biotechnology, Justus Liebig University of Gießen, Gießen, Germany; f LOEWE Centre for Translational Biodiversity Genomics (LOEWE-TBG), Frankfurt am Main, Germany; Georgia Institute of Technology

**Keywords:** *Hermetia illucens*, *Pseudomonas aeruginosa*, antimicrobial agents, antimicrobial peptides, Gram-negative bacteria

## Abstract

Antimicrobial peptides (AMPs) are being explored as alternatives to traditional antibiotics to combat the rising antimicrobial resistance. Insects have proven to be a valuable source of new, potent AMPs with large structural diversity. For example, the black soldier fly has one of the largest AMP repertoires ever recorded in insects. Currently, however, this AMP collection has not yet undergone antimicrobial evaluation or in-depth *in vitro* characterization. This study evaluated the activity of a library of 36 black soldier fly AMPs against a panel of human pathogens (Staphylococcus aureus, Escherichia coli, Pseudomonas aeruginosa, Candida albicans, and Aspergillus fumigatus) and a human cell line (MRC5-SV2). The activity profile of two cecropins (Hill-Cec1 and Hill-Cec10) with potent Gram-negative activity, was further explored by characterizing their hemolysis, time-to-kill kinetics, membrane-permeabilization properties, and anti-biofilm activity. Hill-Cec1 and Hill-Cec10 also showed high activity against other bacterial species, including Klebsiella pneumoniae and multi-drug resistant P. aeruginosa. Both AMPs are bactericidal and have a rapid onset of action with membrane-permeabilizing effects. Hill-Cec1 and Hill-Cec10 were also able to prevent P. aeruginosa biofilm formation, but no relevant effect was seen on biofilm eradication. Overall, Hill-Cec1 and Hill-Cec10 are promising leads for new antimicrobial development to treat critical infections caused by Gram-negative pathogens such as P. aeruginosa.

**IMPORTANCE** With the ever growing antimicrobial resistance, finding new candidates for antimicrobial drug development is indispensable. Antimicrobial peptides have steadily gained attention as alternatives for conventional antibiotics, due to some highly desirable characteristics, such as their low propensity for resistance development. With this article, we aim to upgrade the knowledge on the activity of black soldier fly antimicrobial peptides and their potential as future therapeutics. To achieve this, we have evaluated for the first time a library of 36 synthetically produced peptides from the black soldier fly against a range of human pathogens and a human cell line. Two selected peptides have undergone additional testing to characterize their antimicrobial profile against P. aeruginosa, a clinically important Gram-negative pathogen with a high established resistance. Overall, this research has contributed to the search for new peptide drug leads to combat the rising antimicrobial resistance.

## INTRODUCTION

In the search for new antimicrobials to counteract the rising drug resistance, antimicrobial peptides (AMPs) have gathered a substantial amount of interest. Ever since the discovery of cecropin from the pupae of the silk moth *Hyalophora cecropia* in 1980 (1), insects have steadily gained attention as AMP producers (2–4). Currently, the Antimicrobial Peptide Database reports on 324 AMPs from insect origin, which is the largest repertoire among all animal classes (4, 5). AMPs are small, evolutionary conserved peptides with antimicrobial activity (6). In insects, they are the main effector molecules of the innate immune system and increase their resistance to bacterial infections (2). It is suggested that exposure to pathogens in the insect’s environmental niche is a driving factor behind the evolutionary adaptation of the size and diversity of their AMP repertoire (4, 7).

An insect with an AMP repertoire of remarkable size is the black soldier fly (BSF). The BSF (*Hermetia illucens*, Diptera: Stratiomyidae) expresses over 50 genes encoding putative AMPs, a number that so far has only been recorded for the harlequin *Harmonia axyridis* (8, 9). The larvae of the BSF are saprophagous, feeding preferably on decaying organic matter, including food and agricultural waste, manure, and animal and plant remains (10, 11). Their expansive AMP gene collection has been linked to their survival in these substrates with a high microbial load (8). Apart from their role as defenders against infections, AMPs are also involved in maintaining and shaping the bacterial gut community of the BSF (8, 12). For example, AMP expression in the BSF is diet-dependent and adapts both the feed or substrate microbiota as well as the gut microbiota to allow flexible digestion of the wide range of substrates they encounter in their environment (8, 13). Overall, the use of BSF AMPs could be exploited beyond antimicrobial drug development to applications in the industrial insect farming sector. For instance, AMP addition could help eliminate food pathogens in the insect rearing cycle or stimulate bioconversion of organic waste by *H. illucens* larvae (8). This study, however, focuses on the potential use of AMPs in the development of novel antimicrobial drugs.

To date, the antimicrobial activity of 14 different BSF AMPs has been confirmed *in vitro*, most of these being defensin AMPs (Table 1) (14–22). However, a detailed characterization of their antimicrobial activity is often missing. So far, no *in vitro* antimicrobial evaluation of a full library of BSF AMPs has been performed, leaving much potential of BSF AMPs to be uncovered. This study aims to upgrade the knowledge on the antimicrobial properties of BSF AMPs. This is done first by evaluating a large library of 36 synthetically produced AMPs against a range of pathogenic organisms, and in second by carrying out a deeper *in vitro* characterization of the antimicrobial profile of a selection of two peptides against Pseudomonas aeruginosa.

**TABLE 1:** Overview of antimicrobial peptides of the black soldier fly with *in vitro* verified antimicrobial activity

AMP	AMP family	Study	Activity against	Strength of activity (MIC)^*a*^
DLP4	Defensin	Park et al. (2015)	MRSA^*b*^ S. aureus KCCM 40881 S. aureus KCCM 12256 S. epidermidis KCCM 35494 B. subtilis KCCM 11316	0.59 to 1.17 μM 0.59 to 1.17 μM 1.17 to 2.34 μM 0.59 to 1.17 μM 0.02 to 0.04 μM
		Li et al. (2017)	S. aureus ATCC 25923 S. aureus ATCC 43300 S. aureus ATCC 6538 S. aureus CICC 546 S. suis CVCC 606 *L. ivanovii* ATCC 19119	0.01 μM 0.23 μM 0.47 μM 0.47 μM 1.88 μM 0.12 μM
		Li et al. (2020)	S. aureus CVCC 546 S. epidermis *ATCC* 12228 S. pneumoniae CVCC 2350 S. suis CVCC 3928	3.75 μM 14.99 μM 7.50 μM 3.75 μM
DLP2	Defensin	Li et al. (2017)	S. aureus ATCC 25923 S. aureus ATCC 43300 S. aureus ATCC 6538 S. aureus CICC 546 S. suis CVCC 606 *L. ivanovii* ATCC 19119	0.01 μM 0.12 μM 0.12 μM 0.23 μM 0.93 μM 0.12 μM
DLP3	Defensin	Park et al. (2017)	*MRSA^b^* S. aureus KCCM 40881 S. aureus KCCM 12256 S. epidermis KCCM 25494 E. coli KCCM 11234 P. aeruginosa KCCM 11328	5 μg/mL 5 μg/mL 10 μg/mL 10 μg/mL 10 μg/mL 40 μg/mL
ID13	Defensin	Li et al. (2020)	S. aureus CVCC 546 S. epidermis *ATCC* 12228 S. pneumoniae CVCC 2350 S. suis CVCC 3928	0.95 μM 1.91 μM 0.95 μM 0.95 μM
CLP1	Cecropin	Park et al. (2017)	E. coli KCCM 11234 E. aerogenes KCCM 12177 P. aeruginosa KCCM 11328	0.52 to 1.03 μM 1.03 to 2.07 μM 1.03 to 2.07 μM
Trx-stomoxynZH1a	Cecropin	Elhag et al. 2016)	E. coli S. aureus	15 to 30 μg/mL 27 to 54 μg/mL
HI-attacin	Attacin	Shin et al. (2019)	E. coli KCCM 11234 MRSA^*b*^	No MIC given No MIC given
HiCG13551	IATP	Xu et al. (2020)	E. coli S. aureus S. pneumoniae	No MIC given No MIC given No MIC given
Hidefensin-1	Defensin	Xu et al. (2020)	E. coli	No MIC given
Hidiptericin-1	Diptericin	Xu et al. (2020)	E. coli S. pneumoniae	No MIC given No MIC given
Hill_BB_C6571	Defensin	Moretta et al. (2020)	E. coli	No MIC given
Hill_ BB_C16634	Defensin	Moretta et al. (2020)	E. coli	No MIC given
Hill_BB_C46948	Defensin	Moretta et al. (2020)	E. coli	No MIC given
Hill_BB_C7985	Defensin	Moretta et al. (2020)	E. coli	No MIC given

a MIC, minimum inhibitory concentration.

b MRSA, methicillin-resistant Staphylococcus aureus.

## RESULTS

### Antimicrobial activity of the BSF peptide library.

To identify antimicrobial activity of the AMPs, a screening against one Gram-positive, two Gram-negative, and two fungal species was performed (Table 2, Table S2). One peptide (Hill-Stom2) was not included in the screening due to poor dimethyl sulfoxide (DMSO) solubility. Among the tested peptides, the most promising activity was found within the cecropin family of AMPs. Aside from one peptide (Hill-Cec6), all cecropins showed activity against Escherichia coli and P. aeruginosa at low micromolar concentrations, with minimum inhibitory concentrations (MIC) values ranging from 0.50 μM to 2 μM. Cecropins are α-helical AMPs without cysteine residues and β-sheet motifs (2, 3). Potent activity of insect cecropins with a strict Gram-negative spectrum has been reported for other insect species earlier, such as for *Lucilia sericata* (23, 24). One of the earlier described BSF cecropins (CLP1) has a 95% structure homology with the closest related AMP of our library (Hill-Cec2), and exhibits antibacterial activity in the same concentration range (17). Next, one diptericin (Hill-Dip6) also showed activity against E. coli (MIC of 2 μM), but activity against P. aeruginosa was not recorded. Antibacterial activity against Staphylococcus aureus was reported for three defensins (Hill-Def2a, Hill-Def2b, and Hill-Def4) but was absent for all other AMPs. Interestingly, the free cysteine residues of Hill-Def2a seem crucial for its potent antibacterial activity, as its counterpart with disulfide bridges (Hill-Def2b) needed much higher concentrations (32 μM and 64 μM) for 90% growth inhibition of S. aureus. Finally, no peptide showed signs of activity against Aspergillus fumigatus and Candida albicans within the tested concentration ranges.

**TABLE 2:** IC_50_ values (concentrations causing 50% growth inhibition of the pathogen), MIC values, and MBC values obtained in two individual screenings: the first screening starting at 64μM, the second screening starting at 32 μM^*a*^

		IC_50_ (μM)						MIC (μM)			MBC (μM)		
**AMP**	**Repeat**	**MRC5-SV2**	S. aureusATCC 6538	E. coliATCC 8739	P. aeruginosaATCC 9027	C. albicans B59630	A. fumigatusB42928	S. aureus **ATCC 6538**	E. coliATCC 8739	P. aeruginosaATCC 9027	S. aureusATCC 6538	E. coliATCC 8739	P. aeruginosaATCC 9027
Hill-Knot2	1	**9.73**	>64.00	>64.00	>64.00	>64.00	>64.00	>64.00	>64.00	>64.00			
	2	**3.76**	>32.00	>32.00	>32.00	>32.00	>32.00	>32.00	>32.00	>32.00			
Hill-Knot3	1	>64.00	>64.00	>64.00	**22.63**	>64.00	>64.00	>64.00	>64.00	>64.00			
	2	>32.00	>32.00	>32.00	>32.00	>32.00	>32.00	>32.00	>32.00	>32.00			
Hill-Def2a	1	>64.00	**<0.25**	**35.33**	**32.86**	>64.00	>64.00	**0.25**	>64.00	>64.00	static		
	2	>32.00	**0.19**	>32.00	>32.00	>32.00	>32.00	**2.00**	>32.00	>32.00	static		
Hill-Def4	1	>64.00	**0.50**	>64.00	**32.00**	>64.00	>64.00	**1.00**	>64.00	>64.00	static		
	2	>32.00	**0.70**	>32.00	>32.00	>32.00	>32.00	**8.00**	>32.00	>32.00	static		
Hill-Def2b	1	>64.00	**8.50**	>64.00	>64.00	>64.00	>64.00	**64.00**	32.00	>64.00	static		
	2	>32.00	**13.45**	>32.00	>32.00	>32.00	>32.00	**32.00**	64.00	>32.00	static		
Hill-Cec1	1	>64.00	>64.00	**<0.25**	**<0.25**	>64.00	>64.00	>64.00	**0.25**	**1.00**		1.00	4.00
	2	>32.00	>32.00	**0.21**	**0.20**	>32.00	>32.00	>32.00	**0.50**	**0.50**		0.50	0.50
Hill-Cec2	1	>64.00	>64.00	**0.50**	**0.93**	>64.00	>64.00	>64.00	**1.00**	**4.00**		1.00	16.00
	2	>32.00	**4.36**	**0.31**	**0.76**	>32.00	>32.00	>32.00	**2.00**	**2.00**		2.00	2.00
Hill-Cec3	1	>64.00	>64.00	**0.49**	**0.40**	>64.00	>64.00	>64.00	**1.00**	**4.00**		1.00	16.00
	2	>32.00	>32.00	**0.22**	**0.79**	>32.00	>32.00	>32.00	**0.50**	**2.00**		32.00	2.00
Hill-Cec4	1	37.37	>64.00	**0.50**	**<0.25**	>64.00	>64.00	>64.00	**1.00**	**1.00**		static	16.00
	2	>32.00	>32.00	**0.32**	**0.22**	>32.00	>32.00	>32.00	**2.00**	**2.00**		2.00	2.00
Hill-Cec5	1	>64.00	>64.00	**0.50**	**2.61**	>64.00	>64.00	>64.00	**1.00**	**16.00**		static	static
	2	>32.00	>32.00	**0.23**	**3.60**	>32.00	>32.00	>32.00	**0.50**	**32.00**		0.50	32.00
Hill-Cec7	1	>64.00	>64.00	**0.38**	**0.40**	>64.00	>64.00	>64.00	**1.00**	**4.00**		1.00	16.00
	2	>32.00	>32.00	**0.32**	**0.78**	>32.00	>32.00	>32.00	**2.00**	**2.00**		2.00	2.00
Hill-Cec8	1	>64.00	>64.00	**0.45**	**1.29**	>64.00	>64.00	>64.00	**1.00**	**4.00**		1.00	64.00
	2	>32.00	>32.00	**0.68**	**1.73**	>32.00	>32.00	>32.00	**2.00**	**8.00**		8.00	8.00
Hill-Cec9	1	>64.00	>64.00	**0.43**	**0.38**	>64.00	>64.00	>64.00	**1.00**	**1.00**		1.00	static
	2	>32.00	>32.00	**0.21**	**0.88**	>32.00	>32.00	>32.00	**0.50**	**2.00**		0.50	2.00
Hill-Cec10	1	>64.00	32.74	**<0.25**	**<0.25**	>64.00	>64.00	>64.00	**0.25**	**1.00**		0.25	16.00
	2	>32.00	>32.00	**0.22**	**0.22**	>32.00	>32.00	>32.00	**0.50**	**2.00**		32.00	2.00
Hill-Cec11	1	>64.00	>64.00	**<0.25**	**<0.25**	>64.00	>64.00	>64.00	**1.00**	**1.00**		1.00	4.00
	2	>32.00	>32.00	**0.22**	**0.24**	>32.00	>32.00	>32.00	**0.50**	**2.00**		32.00	8.00
Hill-Cec12	1	>64.00	>64.00	**<0.25**	**<0.25**	>64.00	>64.00	>64.00	**0.25**	**1.00**		1.00	static
	2	19.45	>32.00	**0.32**	**0.23**	>32.00	>32.00	>32.00	**2.00**	**2.00**		8.00	8.00
Hill-Cec13	1	>64.00	>64.00	**<0.25**	**<0.25**	>64.00	>64.00	>64.00	**1.00**	**1.00**		1.00	64.00
	2	>32.00	>32.00	**0.23**	**0.25**	>32.00	>32.00	>32.00	**0.50**	**2.00**		0.50	2.00
Hill-Stom1	1	**11.65**	**58.58**	>64.00	**16.00**	>64.00	>64.00	>64.00	>64.00	>64.00			
	2	**24.42**	>32.00	>32.00	**17.67**	>32.00	>32.00	>32.00	>32.00	>32.00			
Hill-Dip6	1	**4.54**	>32.00	**0.85**	>32.00	>64.00	>64.00	>64.00	**2.00**	>32.00		static	
	2	**7.73**	>32.00	**1.00**	>32.00	>32.00	>32.00	>32.00	**2.00**	>64.00		8.00	

a Peptides with activity < 32 μM against either the cell line (MRC5-SV2) or the microbial reference strains are shown. Static, bacteriostatic activity. Values in bold text indicate that antimicrobial activity could be measured within the tested concentration ranges for the AMP.

### Bactericidal activity of BSF peptide library.

All cecropins exhibited some degree of bactericidal activity against E. coli and P. aeruginosa. The minimum bactericidal concentration (MBC) of the peptides was either equal to their MIC or higher (up to 64 times) and generally, a higher concentration of AMP was required to kill P. aeruginosa compared to E. coli. MBC values were notably variable between the two independent repeat screenings, while for the MIC values only a maximum of a factor two difference was noted between experiments. Thus, the reference strains seem to show a variable susceptibility to the bactericidal mechanism of the cecropin AMPs. Other AMPs with antimicrobial activity, such as the defensins Hill-Def2 and Hill-Def4, did not have a clear bactericidal activity, but rather a bacteriostatic mechanism of action.

### Primary toxicity of BSF peptide library.

Primary peptide toxicity was evaluated using a cell line of human lung fibroblasts (MRC5-SV2) (Table 2). The majority of the tested peptides had no effect on cell viability within the tested concentration ranges. For three AMPs, IC_50_ values below 32 μM were recorded for cell toxicity: Hill-Knot2, a knottin-like peptide, Hill-Stom1, a stomoxyn-like peptide, and Hill-Dip6, a diptericin.

### Structural analysis of the cecropin family.

**FIG 1:**
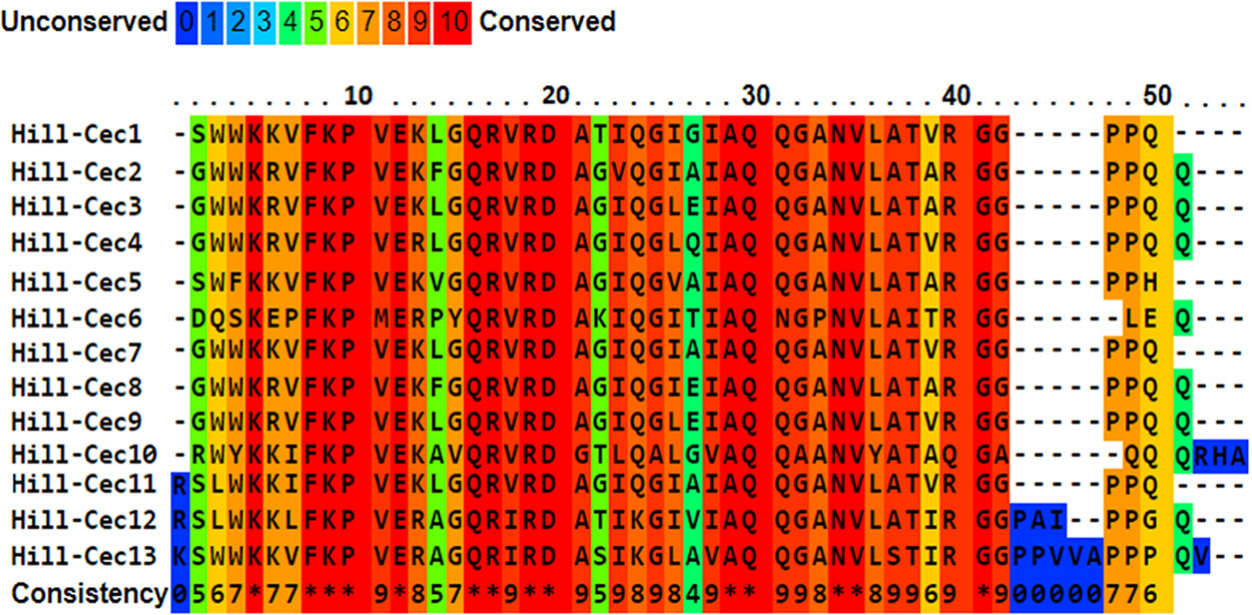
Primary sequence alignment of the BSF AMPs from the cecropin family. The color of the amino acids indicates the extent of conservation among the different peptides. The figure was constructed using the PRALINE software available at https://www.ibi.vu.nl/programs/pralinewww.

To study amino acid conservation among the cecropin family and to compare possible conserved sequence features to the *in vitro* antimicrobial activity, multiple sequence alignment was performed (Fig. 1) (25). Sequence analysis showed strong conservation of most amino acid residues in both the hydrophobic and the polar regions. The absence of a tryptophan residue at the N-terminal region of Hill-Cec6, a reoccurring feature for insect cecropins, could play a role in its lack of antibacterial activity (Table 2, Table S2) (4). Hill-Cec6 also differs from most other cecropins at the C-terminal region, having no proline residues. It also contains more acidic amino acids (AA), giving it a considerably lower net charge (+2) than the other cecropins (+4 to +7).

**FIG 2:**
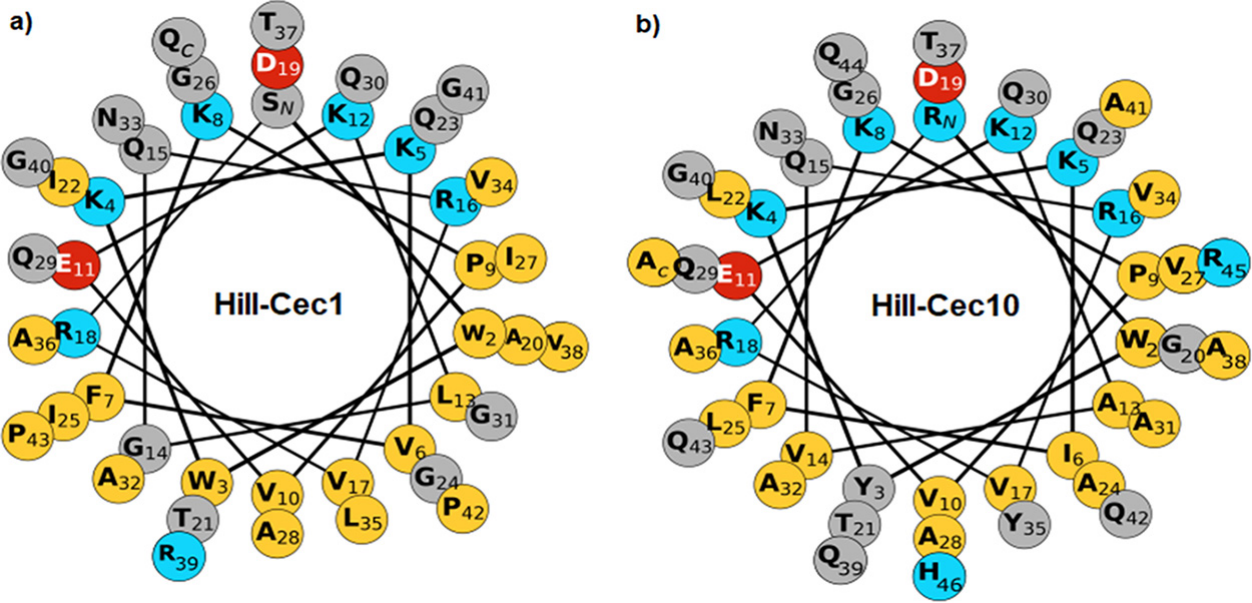
(a) Helical wheel projection of Hill-Cec1. (b) Helical wheel projection of Hill-Cec10. Projections were constructed using the online Galaxy CPT software available at https://cpt.tamu.edu/galaxy-pub. Polar residues with a positive charge are indicated in blue, negatively charged polar residues are red. Uncharged polar amino acids are indicated in gray, and hydrophobic residues have a yellow color.

Two peptides (Hill-Cec1 and Hill-Cec10) from the cecropin family were selected for further *in vitro* characterization. Hill-Cec1 has a charge of +5 and is 44-AA long, whereas Hill-Cec10 has a stronger positive charge of +7 and a length of 47 AA. Both have potent antibacterial activity against the Gram-negative test strains. A helical wheel projection was constructed to visualize the distribution of hydrophobic and hydrophilic amino acids among the helical axis of the peptides (Fig. 2) (26). Both peptides contain a polar region with mainly positively charged residues in opposition to a larger, hydrophobic region.

### Screening of selected cecropins against an extended bacterial panel.

The antibacterial activity of Hill-Cec1 and Hill-Cec10 was further explored against an extended panel of microorganisms (Table 3). As these experiments were performed with a new batch of AMPs, the peptides were also re-evaluated against the previous E. coli and P. aeruginosa test strains. Potent activity comparable to the earlier screening was found for the new AMP batches. Next, P. aeruginosa PAO1, a moderately virulent clinical isolate (27), P. aeruginosa LMG 27650, a multi-drug resistant clinical strain (28), and P. aeruginosa ATCC 15442, an environmental strain (29), were also susceptible to the cecropins. The activity of Hill-Cec1 was comparable with its activity against the non-virulent test strain P. aeruginosa ATCC 9027 (30). The MIC of Hill-Cec10, however, increased by a factor 2 to 4. Hill-Cec10 also required notably higher concentrations for bactericidal activity. Additionally, Hill-Cec1 and Hill-Cec10 were both highly active against K. pneumoniae, another species known to cause critical lung infections (31), with MIC values between 0.25 and 0.5 μM for Hill-Cec1 and between 0.5 and 1 μM for Hill-Cec10. No activity against Burkholderia cenocepacia or Mycobacterium tuberculosis was found within the tested concentration range.

**TABLE 3:** IC_50_ values (concentrations leading to 50% growth inhibition of the pathogen), MIC values (needed for visual absence of bacterial growth), and MBC values obtained in two individual screenings of the selected cecropins Hill-Cec1 and Hill-Cec10 performed at concentrations starting at 32 μM

		IC_50_ (μM)		MIC (μM)		MBC (μM)	
Strain	Repeat	Hill-Cec1	Hill-Cec10	Hill-Cec1	Hill-Cec10	Hill-Cec1	Hill-Cec10
E. coli ATCC 8739	1	0.15	0.73	0.5	1	0.5	1
	2	0.15	0.63	0.25	1	0.25	32
P. aeruginosa ATCC 9027	1	0.64	0.75	1	1	1	8
	2	0.37	0.75	0.5	2	16	16
P. aeruginosa ATCC 15692 (PAO1)	1	0.77	2.48	1	8	4	16
	2	0.39	0.76	0.5	2	0.5	16
P. aeruginosa LMG 27650 (MDR)^*a*^	1	1.34	2.96	2	4	4	32
	2	0.66	3.35	1	8	16	16
P. aeruginosa ATCC 15442	1	0.73	2.82	1	4	4	16
	2	0.38	2.71	1	4	8	32
K. pneumoniae ATCC 13883	1	0.36	0.75	0.5	1	2	4
	2	0.18	0.31	0.25	0.5	4	8
B. cenocepacia LMG 16656	1	>32	>32	>32	>32		
	2	>32	>32	>32	>32		
M. tuberculosis ATCC 25177 (H37Ra)	1	>32	>32	>32	>32		
	2	>32	>32	>32	>32		

a MDR, multi-drug-resistant.

### Hemolysis analysis.

In addition to the primary toxicity screening against human fibroblasts, a hemolytic assay was performed using human red blood cells. The outer membrane leaflets of red blood cells are rich in sialic acid residues, which gives them a lower negative charge than other mammalian cell types (32, 33). This negative charge makes them prone to interactions with the positively charged AMPs. Hence, a hemolysis analysis is often routinely included in AMP research. Apart from cationicity, hydrophobicity is also positively correlated with the hemolytic capacity of AMPs (34). Both cecropins showed hemolysis of less than 10% at the highest concentration tested of 64 μM. These results predict low hemolysis *in vivo*. However, results will need to be confirmed later on *in vivo*, as the experimental conditions differ from the protein-rich environment of whole blood (35). Results of the hemolysis analysis were in line with the bio-informatical predictions of HemoPred which identified the two cecropins as “nonhemolytic” (36).

### Time-to-kill analysis.

**FIG 3:**
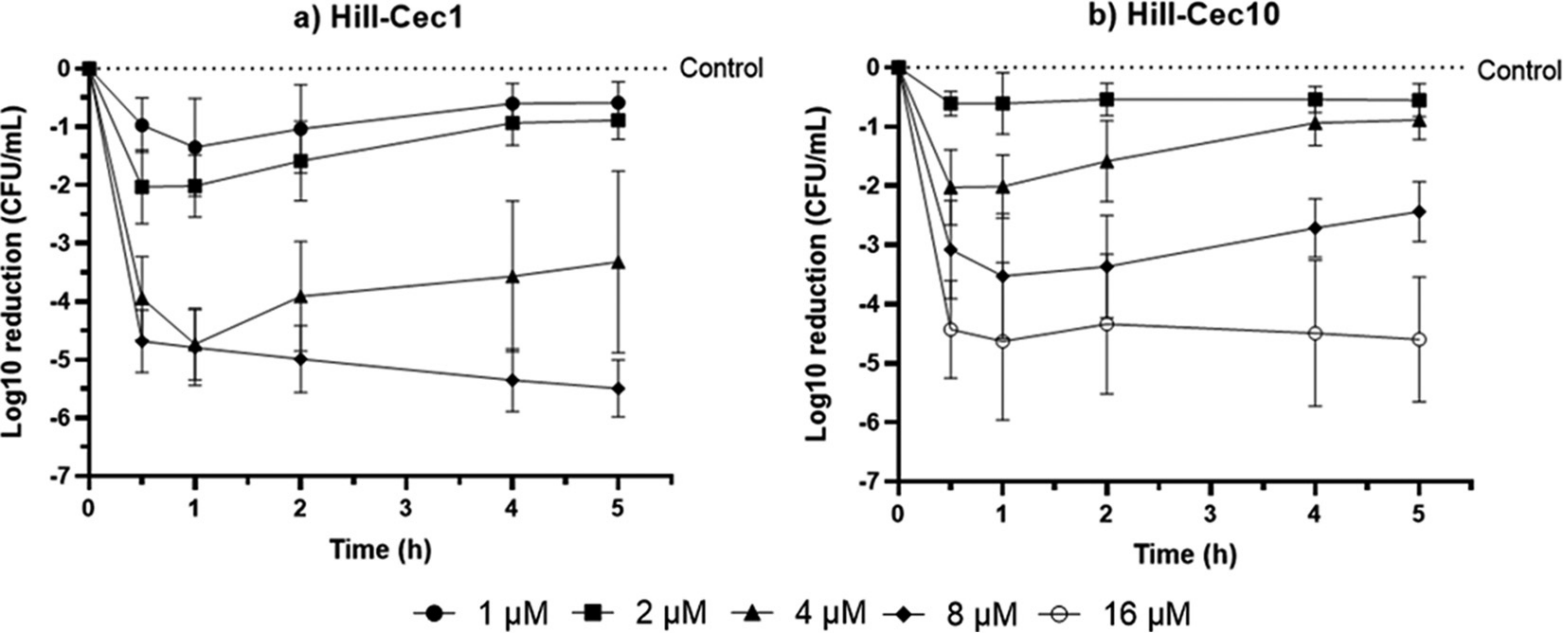
Time-to-kill curves showing the log_10_ reductions in P. aeruginosa ATCC 9027 caused by the selected cecropins. (a) Killing kinetics of Hill-Cec1 at 1 μM, 2 μM, 4 μM, and 8 μM. (b) Killing kinetics of Hill-Cec10 at 2 μM, 4 μM, 8 μM, and 16 μM. Graphs represent the mean of five independent experiments.

To investigate the onset of action and the bactericidal activity of the peptides, a time-to-kill analysis was performed. Both cecropins have a rapid onset of action and cause growth inhibition of P. aeruginosa within 30 min (Fig. 3a and b). To obtain bactericidal activity (≥3-log_10_ reduction compared with the growth control), both AMPs require at least a concentration of four times their MIC values. At 4 μM (4 × MIC), Hill-Cec1 showed a rapid bactericidal activity, leading to a log_10_ reduction of 4.74 ± 0.55 after 1 h. However, afterwards there was a clear increase in P. aeruginosa growth, and at 5 h, Hill-Cec1 had variable activity throughout the different experiments, causing on average a 3.32 ± 1.40 log_10_ reduction. In contrast, a higher Hill-Cec1 concentration of 8 μM (8 × MIC) consistently achieved bactericidal activity at all time points tested, leading to a log_10_ reduction in bacteria of 5.50 ± 0.44 after 5 h. For Hill-Cec10, a similar pattern was observed. The highest concentration of 16 μM (8 × MIC) always caused at least a 3-log_10_ reduction of bacteria, although the exact amount of bacterial killed varied throughout the independent experiments, leading to a log_10_ reduction of 4.60 ± 0.94 after 5 h. At 8 μM (4 × MIC), the AMP was bactericidal up until 1 h, but was not able to sustain this killing effect for the next time points tested (log_10_ reduction of 2.44 ± 0.46 after 5 h).

Important to mention is the variability in bactericidal activity noticed in the time-to-kill experiments. This variability was also seen in our earlier MBC experiments, and is suspected to be caused by natural variation in the peptides’ MIC and MBC values in-between experiments.

### Inhibition of biofilm formation.

**FIG 4:**
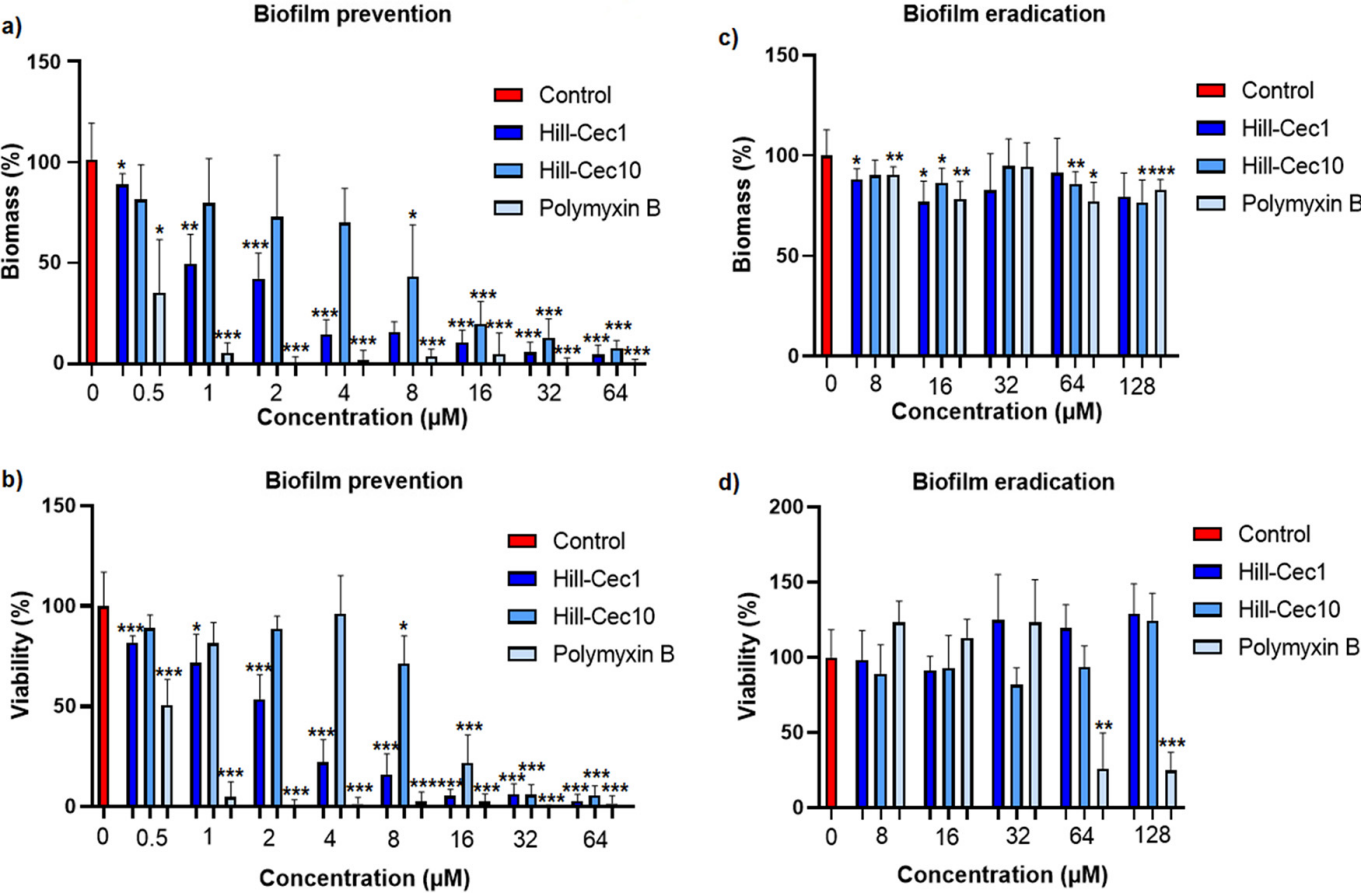
Anti-biofilm activity of cecropins Hill-Cec1 and Hill-Cec10 against P. aeruginosa ATCC 15442. (a) Effect of Hill-Cec1 and Hill-Cec10 on P. aeruginosa biofilm formation as measured by biofilm mass. (b) Effect of Hill-Cec1 and Hill-Cec10 on P. aeruginosa biofilm formation as measured by biofilm viability. (c) Effect of Hill-Cec1 and Hill-Cec10 on preformed P. aeruginosa biofilms as measured by biofilm mass. (d) Effect of Hill-Cec1 and Hill-Cec10 on preformed P. aeruginosa biofilms as measured by biofilm viability. Bars represent the mean ± SD of three independent experiments. *, *P* ≤ 0.05; **, *P* ≤ 0.01; ***, *P* ≤ 0.001.

As biofilm formation of P. aeruginosa strains contributes to their tolerance and resistance to antibiotics (37), effects of the cecropin AMPs on biofilm formation was investigated. P. aeruginosa ATCC 15442 was used as it showed less variability in biofilm formation in our assay than P. aeruginosa PAO1, as observed in preliminary experiments (data not shown). Both peptides exhibited a concentration-dependent inhibition of biofilm formation. For Hill-Cec1, a statistically significant decrease, compared with the untreated control, of biofilm mass (*P* < 0.05) and viability (*P* < 0.001) at sub-MICs (0.5 μM) was noted. Hill-Cec1 achieved 50% reduction in biofilm mass at a concentration close to its MIC (IC_50_ of 1.3 ± 0.57 μM), and 50% reduction of biofilm viability at supra-MICs (IC_50_ of 2.1 ± 0.52 μM) (Fig. 4a). For Hill-Cec10, however, higher concentrations were needed to obtain 50% reduction or more of biofilm mass (IC_50_ of 7.5 ± 3.5 μM) or biofilm viability (IC_50_ of 11 ± 1.7 μM) (Fig. 4b). The difference in bactericidal concentrations between both cecropins could explain their difference in inhibition of biofilm formation.

### Biofilm eradication.

Hill-Cec1 and Hill-Cec10 were not able to eradicate a preformed P. aeruginosa biofilm. Only marginal reductions of biofilm mass were seen for Hill-Cec1 (between 9% and 23%) and Hill-Cec10 (between 10% and 23%), and the decrease was not concentration-dependent (Fig. 4c). No significant effect on biofilm viability compared with the untreated control was detected (Fig. 4d). In comparison, the reference polymyxin B, a peptide antibiotic, shows over 70% reduction in viability at concentrations of 64 μM and 128 μM.

### Membrane permeabilization and disruption.

**FIG 5:**
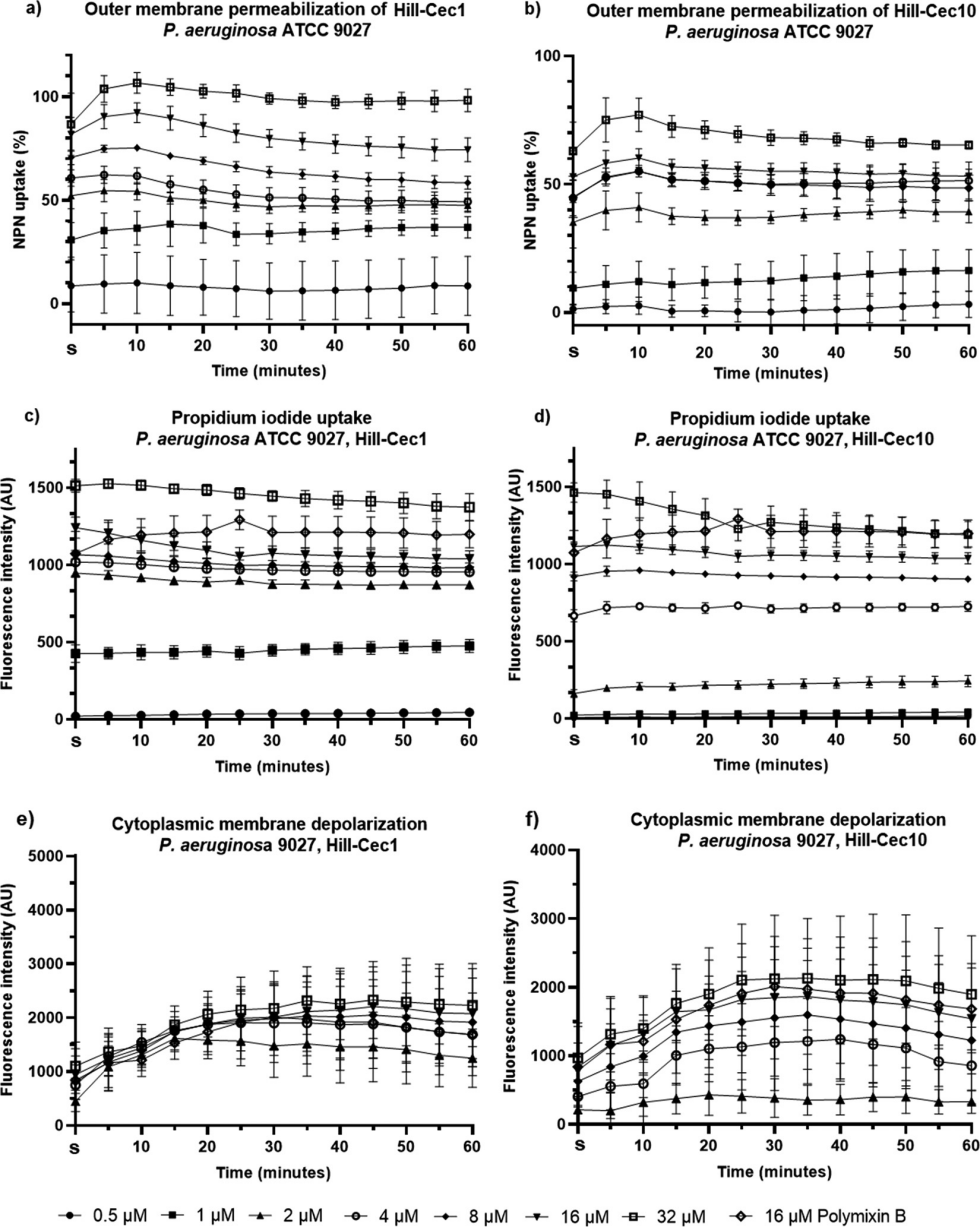
Membrane activity of Hill-Cec1 and Hill-Cec10. (a) Outer membrane (OM) permeabilization *of* P. aeruginosa caused by Hill-Cec1 measured with N-phenyl-naphthylamine (NPN). (b) OM permeabilization *of* P. aeruginosa caused by Hill-Cec10 measured with NPN. NPN uptake is expressed as a percentage of the maximal uptake recorded with a high dose (16 μM) of polymyxin B. (c) Fluorescence caused by propidium iodide (PI) uptake in P. aeruginosa after addition of Hill-Cec1. (d) Fluorescence caused by PI uptake in P. aeruginosa after addition of Hill-Cec10. Values were normalized with the negative control. (e) Fluorescent signal of 3,3′-dipropylthiadicarbocyanine iodide (diSC_3_(5)) as an indicator of cytoplasmic membrane depolarization of P. aeruginosa caused by Hill-Cec1. (f) Fluorescent signal of diSC_3_(5) as an indicator of cytoplasmic membrane depolarization of P. aeruginosa caused by Hill-Cec10. Fluorescent signals were normalized with the negative control. AU, arbitrary units; S, start of measurement immediately after dye exposure.

Both cecropins are able to permeabilize the cell membranes of P. aeruginosa, as confirmed by the N-phenyl-naphthylamine (NPN) and propidium iodide (PI) uptake assays (Fig. 5). NPN, a hydrophobic probe, is normally excluded from bacterial membranes. It will, however, accumulate in the outer membrane (OM) of Gram-negative bacteria when the barrier is compromised, leading to an increase in fluorescence (38, 39). NPN uptake in the OM occurs within the first 5 min, after which the fluorescent signal stabilizes (Fig. 5a and b). The NPN uptake is concentration-dependent and reaches a maximum of 100% (compared with 16 μM polymyxin B) at 32 μM for Hill-Cec1, and a maximum of 75% at 32 μM for Hill-Cec10. Supra-MICs are needed to reach 50% or more NPN uptake. Upon treatment with PI, a sharp increase in fluorescence is also seen immediately after exposure, after which the signal quickly stabilizes (Fig. 5c and d). PI is a DNA intercalating dye, which is not able to traverse intact bacterial membranes (40). When the membranes are permeabilized, PI is able to reach the cytoplasm where it binds to nucleic acids, which increases its fluorescent signal (38). PI fluorescence is therefore an indicator of inner membrane (IM) permeabilization. The PI uptake is concentration-dependent for both cecropins. At high concentrations (32 μM) the membrane permeabilization exceeds that of a high dose of polymyxin B (16 μM). The NPN and PI uptake assays show that Hill-Cec1 and Hill-Cec10 can permeabilize both the OM and IM of P. aeruginosa. The majority of AMPs with a characterized mechanism of action work by decreasing the integrity of the bacterial membranes through, for example, pore formation or complete membrane lysis (41). As the increase in fluorescence for both the NPN and PI uptake occurs rapidly (within 5 min) and is present at non-lethal concentrations, the membrane permeabilization is likely directly linked to the mechanism of action of the BSF AMPs, and not a secondary effect of the bacteria dying through other, non-related intracellular mechanisms (42).

### Cytoplasmic membrane depolarization.

DiSC3(5), a voltage-sensitive cationic dye, was used to study cytoplasmic membrane depolarization. Under normal conditions, the dye is concentrated in the (hyper)polarized cytoplasmic membrane, leading to self-quenching of its fluorescence. Upon depolarization, however, diSC3(5) is released into the cytoplasm causing an increase in fluorescence (43). Both Hill-Cec1 and Hill-Cec10 cause cytoplasmic membrane depolarization of P. aeruginosa, as indicated by the increase in diSC3(5) fluorescence (Fig. 5e and f). The depolarization is largely concentration-dependent, although more outspoken for Hill-Cec10. At high concentrations, cytoplasmic depolarization exceeds that of polymyxin B (16 μM), especially for Hill-Cec1. As the PI uptake assay showed that the cecropins permeabilize the IM, it is possible that the membrane depolarization is largely caused by membrane damage.

## DISCUSSION

### Biological function of cecropins in the black soldier fly.

As a decomposer of biowaste, the BSF larva lives in close contact with potentially hazardous microorganisms (16). The evolutionary expansion and diversification of the BSF AMP repertoire could explain their successful colonization of these microbe-rich substrates (8). After a broad antimicrobial screening of a library of BSF peptides, we found the majority of the activity situated in the cecropin family. Apart from Hill-Cec6, they all showed strong activity against the Gram-negative test strains at low micromolar concentrations. Cecropin AMPs are widely distributed among the insect orders, and have so far been identified in the Coleoptera, Diptera, and Lepidoptera. It is suggested, however, that they are produced by all holometabolous insects with the exception of Hymenopteran species (4). The amount and diversity of cecropins found in these insects hints at more preserved functions of these AMPs. In the BSF, AMPs are involved in maintaining and shaping the bacterial gut communities and maintaining eubiosis in the gut (8, 12), but cecropins could have additional biological roles as well. For example, the Aedes aegypti cecropin B has been shown to be involved in the formation of the cuticle of adult mosquitoes (44). Knockdown of the cecropin B gene in the pupae led to high mortality, deformed adults, and impaired cuticle lamellae with disorganized chitin fibrils (44). It is suggested that cecropin B works through upregulating the expression of prophenoloxidases, which are involved in cuticle formation. Prophenoloxidases also play a crucial role in insect immunity. The enzymes are, for example, involved in the induction of the melanization process, which leads to encapsulation of invading pathogens (45, 46).

### Potential of black soldier fly cecropins in drug development.

The activity against Gram-negative pathogens of cecropins has made them compounds of interest for new antimicrobial drug development (41). In this study, we selected two cecropin AMPs, Hill-Cec1 and Hill-Cec10, for *in vitro* characterization. In line with other cecropins, they have an alpha-helical, amphiphatic domain and a strong positive net charge. As AMPs are known to be toxic to human cells due to unspecific membrane interactions, we studied their effect on lung fibroblasts and erythrocytes (35). Both peptides showed no signs of *in vitro* hemolysis or cytotoxicity. The antimicrobial activity of the cecropins was further explored, and apart from the E. coli and P. aeruginosa test strains, the cecropins were also active against other P. aeruginosa species, including a multidrug-resistant strain, and K. pneumoniae.

Next, we characterized the antimicrobial profile of Hill-Cec1 and Hill-Cec10 against P. aeruginosa more in-depth. P. aeruginosa is an opportunistic pathogen that is a major cause of nosocomial infections (47). Vulnerable patients, such as cystic fibrosis and burn wound patients, are especially at risk of Pseudomonas infections (47, 48). Eradication of P. aeruginosa has become increasingly difficult due to the rise of multidrug-resistant strains (49). AMPs with activity against these drug-resistant strains could be valuable in the treatment of these critical infections (41). Both cecropins are able to kill P. aeruginosa bacteria within the first 30 min of exposure. However, to maintain bactericidal activity, supra-MICs are needed (4 to 8 μM for Hill-Cec1, 16 μM or higher for Hill-Cec10). Bactericidal activity has been linked to membrane permeabilization for other cecropins (50, 51). Indeed, the NPN uptake assay confirmed that Hill-Cec1 and Hill-Cec10 are able to permeabilize the OM of P. aeruginosa. In line with the killing kinetics, the peptides are able to disrupt the OM fast (within 5 min), but need supra-MICs (2 μM for Hill-Cec1, 4 μM for Hill-Cec10) to achieve at least 50% NPN uptake. The PI and diSC_3_(5) assays show that both cecropins are also able to target the inner, cytoplasmic membrane. Membrane disruption by AMPs is one of the main mechanisms that prevents pathogens to develop resistance (52, 53). The combination of their activity against multi-drug resistant Pseudomonas, their low cytotoxicity, their fast killing time and the membrane disruptive mechanism of action, make Hill-Cec1 and Hill-Cec10 candidates for new antipseudomonal drug leads to treat acute infections, such as skin or lung infections (54, 55).

Finally, anti-biofilm activity of the cecropins was studied. Biofilm formation of P. aeruginosa can lead to persistent infections that respond poorly to antibiotic treatment. The biofilm matrix can slow antibiotic penetration, while sessile bacteria enter into a dormant state characterized by a high antimicrobial tolerance (37). Two strategies to combat biofilms are (i) the prevention of biofilm formation by either inhibiting bacterial adhesion or by reducing initial bacterial growth, and (ii) the eradication of mature biofilms (56). Both strategies were investigated within this study. Hill-Cec1 and Hill-Cec10 showed a significant decrease in biofilm mass and viability when they were added immediately to planktonic P. aeruginosa bacteria. Bactericidal concentrations of the cecropins were able to obtain 75% or more reduction of biofilm formation, indicating that the majority of the inhibitory action of the peptides is directly linked to their activity on the planktonic bacteria. Cecropins with inhibitory activity can be useful to prevent biofilms after surgery or as a coating on medical devices (57–59). When the peptides were added to 16-h mature biofilms, no significant effect on biofilm viability was observed at concentrations up to 128 μM. Possibly, their high positive charge leads to electrostatic interactions with anionic polymers in the biofilm matrix, causing them to be trapped (60). For some other cecropins, however, activity against preformed biofilms of Gram-negative pathogens has been reported (61, 62).

### Importance in industrial black soldier fly larvae production.

Apart from antimicrobial drug development, there is substantial interest in exploiting active AMPs toward other applications and industries. AMPs could, for example, be used as additives in the agriculture, food, and feed industries (63–65). BSFL are being studied in waste management and recycling in livestock farming, for example in the hygienization of manure (66–70). The reduction of pathogens in these highly contaminated substrates by the BSFL is partially attributed to the larvae’s production of AMPs (70). Furthermore, AMPs could also be of use in the insect industry itself. Insect farming is a flourishing sector (71), and the application of AMPs in BSF farming could help to lower the bioburden, including the number of human pathogens, present in the substrate during rearing, the insect biomass and the leftover frass (72, 73). However, in order to develop these AMPs into usable additives, more research needs to be done on the stability of these peptides, for example in the substrate, and possible AMP formulations.

### Concluding remarks.

Overall, our selected cecropin peptides have shown promising activity against Gram-negative pathogens, such as P. aeruginosa. This leaves potential for various industrial applications including antimicrobial drug development. As insect AMPs are studied as alternative treatments for infections, such as skin, eye, and lung infections, these BSF cecropins expand the library of peptide templates usable for antimicrobial drug development (54). However, many more aspects, such as synergy with conventional antibiotics, of these cecropin AMPs remain to be characterized (74, 75). Moreover, further development will have to address obstacles commonly associated with peptide-based drugs, including poor metabolic stability (4, 76).

## MATERIALS AND METHODS

### Antimicrobial peptides.

In a previous study, genes were identified in the *H. illucens* transcriptome encoding for putative AMPs (8). All AMPs of this study that could be produced synthetically were produced by solid-phase peptide synthesis and purified by either COVALAB (Bron, France), Proteogenix (Schiltigheim, France), or Genscript (Leiden, the Netherlands). Liquid chromatography-mass spectrometry was used to determine the peptide purity. Peptide type, amino acid sequence, purity, C-terminal modifications, isoelectric point, and molecular weight are summarized in Table S1. For the experiments, peptides were dissolved in DMSO (Acros Organics) at a concentration of 10 mM and further diluted in sterile demineralized water.

### Bacterial isolates and culture conditions.

P. aeruginosa ATCC 9027, P. aeruginosa ATCC 15442, P. aeruginosa ATCC 15692 (PAO1), E. coli ATCC 8739, S. aureus ATCC 6538, K. pneumoniae ATCC 13883, and M. tuberculosis ATCC 25177 (H37Ra) were obtained from the ATCC (American Type Culture Collection, Manassas, VA, USA). B. cenocepacia LMG 16656, P. aeruginosa LMG 27650, A. fumigatus B42928, and C. albicans B59630 were obtained from the Belgian Coordinated Collections of Microorganisms. Bacterial strains (except M. tuberculosis) were cultured in Mueller-Hinton broth (MHB; Difco) and on Mueller-Hinton agar (MHA; Sigma-Aldrich) or tryptic soy agar (TSA; Sigma-Aldrich). M. tuberculosis was grown in complete Middlebrook 7H9 (Sigma-Aldrich) medium. Fungal species were grown in Roswell Park Memorial Institute (RPMI; Gibco) medium.

### Antimicrobial activity assay.

To detect antimicrobial activity of the BSF AMPs, the peptide library underwent screening against a panel of microorganisms, consisting of S. aureus ATCC 6538, E. coli ATCC 8739, P. aeruginosa ATCC 9027, C. albicans B59630, and A. fumigatus B42928. Serial dilutions of the peptides were prepared from the DMSO stock solutions in sterile demineralized water in 96-well plates using an automated liquid-handling workstation (Beckman Coulter Biomek 3000) in end volumes of 10 μL. The final in-plate concentration of DMSO was <1%. Peptide concentrations ranged from 64 μM to 0.25 μM for the first evaluation, and from 32 μM to 0.016 μM for the independent repeat. As references, doxycycline (Sigma-Aldrich; S. aureus, E. coli, P. aeruginosa), flucytosine (Sigma-Aldrich*;* C. albicans), and econazole (Sigma-Aldrich*;* A. fumigatus) were used. Suspensions of a starting inoculum of 5 × 10^3 CFU/mL (E. coli, S. aureus, C. albicans, A. fumigatus) and 5 × 10^4 CFU/mL (P. aeruginosa) were prepared in MHB (bacterial species) or RPMI medium (fungal species) and 190 μL was added to the 96-well plates. Plates were incubated for 16 h (E. coli, S. aureus, P. aeruginosa), 24 h (C. albicans), or 48 h (A. fumigatus) at 37°C. After incubation, read-out of the antimicrobial activity was performed using a resazurin assay. Then, 20 μL of a 0.01% (wt/vol) resazurin (Sigma-Aldrich) solution was added to each well and plates were incubated for 15 min (S. aureus), 30 min (E. coli), 45 min (P. aeruginosa), 4 h (C. albicans), or 17 h (A. fumigatus) to allow resazurin reduction to take place. Fluorescence was read using a microplate reader (Telix) at λ_excitation_ = 550 nm and λ_emission_ = 590. The results were used to calculate the IC_50_ value, defined as the concentration of peptide causing 50% microbial growth inhibition. MIC values were determined visually. Medium in the wells with no visual growth was plated on MHA to detect the MBC, defined as ≥3 log reduction compared with the growth control.

### Cytotoxicity screening of the peptide library.

To determine early signs of peptide toxicity, the library was tested against the MRC5-SV2 cell line of human, embryonic lung fibroblasts (Sigma-Aldrich). Peptides were serially diluted in 96-well plates as described earlier at concentrations ranging from 64 μM to 0.25 μM for the first screening, and from 32 μM to 0.016 μM for the independent repeat. Tamoxifen (Sigma-Aldrich) was included as a reference compound. After preparation of the test plates, cell suspension (190 μL) of 1.5 × 10^5 cells/mL in complete minimum essential medium (Gibco) was added to the peptides. Plates were incubated for 72 h at 37°C in a 5% CO_2_ incubator (Binder). Afterwards, 50 μL of a 0.01% (wt/vol) resazurin (Sigma-Aldrich) solution was added to the wells to detect cell viability. After 4 h of incubation, fluorescence was read using a microplate reader (Telix) at λ_excitation_ = 550 nm and λ_emission_ = 590 and the IC_50_ values were calculated.

### Evaluation of selected cecropins against an extended bacterial panel.

To further investigate the antimicrobial spectrum of the selected AMPs, *in vitro* antimicrobial screening against additional microorganisms was performed. The extended panel of microbes included P. aeruginosa PAO1, P. aeruginosa LMG 27650 (a multidrug-resistant strain), P. aeruginosa ATCC 15442, K. pneumoniae ATCC 13883, B. cenocepacia LMG 16656, *and* M. tuberculosis H37Ra. Peptides were serially diluted in sterile demineralized water in 96-well plates using an automated liquid-handling workstation as described above. Bacterial suspensions were prepared at a concentration of 10^4 CFU/mL (K. pneumoniae), 5 × 10^4 CFU/mL (P. aeruginosa), 10^5 CFU/mL (B. cenocepacia), or 5 × 10^5 CFU/mL (M. tuberculosis). In addition, 190 μL of this suspension was mixed with the diluted peptides. For the M. tuberculosis screen, the outer wells of the test plates were filled with 200 μL of demineralized water. As references, doxycycline (Sigma-Aldrich; P. aeruginosa PAO1, P. aeruginosa ATCC 15442, K. pneumoniae), polymyxin B sulfate (Sigma-Aldrich; P. aeruginosa LMG 27650), moxifloxacine (Sigma-Aldrich; B. cenocepacia), and isoniazide (Sigma-Aldrich; M. tuberculosis) were used. Test plates were incubated at 37°C for 16 h (P. aeruginosa, K. pneumoniae), 48 h (B. cenocepacia), or 7 days (M. tuberculosis) and read-out of the antimicrobial activity was performed with a resazurin assay as described earlier. Plates with resazurin were incubated 15 min (K. pneumoniae), 30 min (P. aeruginosa), 4 h (B. cenocepacia), or 1 day (M. tuberculosis). The fluorescent signal was read using a microplate reader (Promega) at λ_excitation_ = 550 nm and λ_emission_ = 590 and the IC_50_ values were calculated.

### Hemolysis analysis.

For the analysis of hemolysis, fresh human whole blood was collected in tubes containing 30 units of heparin (77). Afterwards, the blood was centrifuged (1,000 × *g*, 5 min) and washed until the supernatant was clear. The erythrocyte pellet was diluted in phosphate-buffered saline (PBS; Gibco) to obtain a 2% red blood cell suspension. Next, 150 μL of serially diluted AMPs in PBS were added to microcentrifuge tubes and mixed with 150 μL of the red blood cell suspension. Furthermore, 0.1% Triton-X (Sigma-Aldrich) and PBS were used as positive and negative controls, respectively. The samples were incubated at 37°C for 1 h. Afterwards, the tubes were centrifuged at 1,000 × *g* for 5 min and 200 μL of the supernatant was transferred to a 96-well plate. Absorbance was measured with a microplate reader (Promega) at λ = 570 nm to detect hemoglobin release. The hemolysis analysis was carried out twice. In addition, hemolysis was predicted using the online software HemoPred (36).

### Time-to-kill analysis.

To investigate the bactericidal kinetics of the selected AMPs, a time-to-kill analysis was performed as described previously by Mascio et al. (78). Briefly, peptide serial dilutions were prepared in 96-well plates (10 μL) and mixed with 190 μL of a P. aeruginosa ATCC 9027 culture of an optical density measured at a wavelength of 600 nm (OD_600_) of 0.1. Plates were incubated at 37°C and at selected time points (0 h, 0.5 h, 1 h, 2 h, 4 h, and 5 h), aliquots were removed and serially diluted in PBS. The dilutions were plated on TSA and incubated overnight at 37°C. Afterwards, bacterial viability was assessed by performing a standard plate count. Bactericidal activity was defined as a ≥3-log reduction compared with the untreated bacterial control. To avoid carry-over effect of the peptides, the undiluted sample was not plated, leading to a quantification limit of 10^3 CFU/mL (78). Bacterial counts were calculated for each AMP concentration at each time point using the average plate count. Afterwards, log_10_ reductions of the bacterial counts were calculated using the following formula: log_10_ reduction = log_10_ (viable microorganisms without AMP treatment/viable microorganisms with AMP treatment). Next, the average log_10_ reductions and the standard deviations of five independent experiments were calculated and visualized using GraphPad Prism 8.

### Inhibition of biofilm formation.

To investigate the effect of the AMPs on biofilm formation, serially diluted peptides were added *in duplo* to 96-well plates, with final concentrations ranging from 64 to 0.5 μM. Next, a P. aeruginosa ATCC 15442 culture of approximately 10^6 CFU/mL was added. After 16 h of incubation at 37°C and shaking at 110 rpm (New Brunswick Innova 4300), a resazurin assay was used as described by Gilbert-Girard et al. to determine the viability of the bacterial cells in the biofilm (79). After incubation, the growth medium was removed from the well plates and the bacterial biofilms were washed once with PBS to remove remaining planktonic cells. Afterwards, 200 μl of a 20 μM resazurin solution was added to each well and the plates were incubated 4 h at 37°C with agitation at 110 rpm (New Brunswick Innova 4300). The fluorescence of the wells was measured at λ_excitation_ = 550 nm and λ_emission_ = 590 using a microplate reader (Promega). Afterwards, a crystal violet staining was applied to determine the biomass of the biofilms (79). The resazurin solution was removed from the well plates and 200 μL of 100% ethanol (VWR Chemicals) was added to fix the biofilms. After 15 min of incubation, the ethanol was discarded from the wells and the plates were left to air dry for 30 min. Afterwards, 200 μL of a 0.023% (wt/vol) crystal violet (Merck) solution was added to the biofilms. The staining solution was removed after 5 min and the biofilms were washed with PBS. After air-drying for 10 min, the crystal violet stain was solubilized in 200 μL of 100% ethanol. Absorbance was measured at λ = 595 nm with a microplate reader (Promega) and, where possible, IC_50_ values were calculated. The experiment was carried out three times. Polymyxin B sulfate (Sigma-Aldrich) was included as a reference.

### Biofilm eradication (postexposure).

In addition to biofilm formation, the effect of the peptides on preformed biofilms was investigated to measure the amount of biofilm eradication. P. aeruginosa ATCC 15442 biofilms were grown in 96-well plates as described above without the addition of AMPs. After overnight incubation, the medium was removed and 10 μL of serially diluted peptides were added *in duplo* to the plates at concentrations ranging from 128 μM to 0.5 μM (final concentration). New MHB was added and the well plates were incubated again for 16 h at 37°C and shaking at 110 rpm (New Brunswick Innova 4300). The next day, bacterial viability and biomass were determined with a resazurin assay and crystal violet assay as described earlier. The experiment was carried out three times. Polymyxin B sulfate was included as a reference.

### Inner membrane permeabilization by the propidium iodide uptake assay.

To investigate the IM damage induced by the selected AMPs, a PI (Sigma-Aldrich) uptake assay was performed, adapted from Dassanayake et al. (80). P. aeruginosa ATCC 9027 was grown until the mid-log phase in MHB. The bacteria were centrifuged (3,000 × *g*, 15 min) and resuspended in 5 mM HEPES (Sigma-Aldrich) buffer (pH 7.4) to an OD_600_ of 0.5. Next, 50 μL of serially diluted peptides were added *in duplo* to a 96-well plate and mixed with a 150 μL bacterial suspension containing 4 μM PI (final in-plate concentration of 3 μM). Peptide test concentrations ranged from 32 μM to 0.25 μM. A high concentration of polymyxin B sulfate (16 μM) was implemented as a positive control. Fluorescence was measured every 5 min during 1 h using a microplate reader (Promega) at λ_excitation_ = 530 nm and λ_emission_ = 620. Data were normalized based on the fluorescent signal in the presence of 50 μL HEPES buffer and 150 μL PI bacterial suspension. The assay was carried out in triplicate.

### Outer membrane permeabilization with N-phenyl-naphthylamine.

N-phenyl-naphthylamine (NPN; Tokyo chemical industry [TCI]) was used to study the permeabilization of the OM as described by Helander et al. (81). Briefly, P. aeruginosa ATCC 9027 was grown until the mid-log phase in MHB. Afterwards, the cells were centrifuged (3,000 × *g*, 15 min) and resuspended in 5 mM HEPES buffer (pH 7.4) to an OD_600_ of 0.5. Next, 50 μL of peptides were added in duplo to a 96-well plate (final concentrations ranging from 32 μM to 0.25 μM). As a positive control, 16 μM polymyxin B was included. The peptides were mixed with 50 μL of a 40 μM NPN solution and 100 μL of P. aeruginosa suspension. Fluorescence was measured every 5 min during 1 h using a microplate reader (Promega) with λ_excitation_ = 350 nm and λ_emission_ = 420. Data were normalized based on the fluorescent signal in the presence of 50 μL HEPES buffer, 50 μL NPN, and 100 μL bacteria. Experiments were carried out in triplicate.

### Cytoplasmic membrane depolarization assay.

To study the effects of the peptides on the membrane potential, 3,3′-dipropylthiadicarbocyanine iodide (diSC3(5); TCI) was used as described by Kwon et al. (82). P. aeruginosa ATCC 9027 was grown until the mid-log phase in MHB. Afterwards, the cells were centrifuged (3,000 × *g*, 15 min), washed and resuspended in 5 mM HEPES buffer (pH 7.4) supplemented with 20 mM glucose and 100 mM KCl to an OD_600_ of 0.2. DiSC3(5) was added to the P. aeruginosa suspension at a concentration of 1.33 μM (final in-plate concentration of 1 μM), and the mixture was left to stand for 1.5 h to stabilize the fluorescent signal. Serially diluted peptides (50 μL) were added in duplo to 96-well plates (final concentrations ranging from 32 μM to 0.25 μM) and mixed with 150 μL of bacteria with diSC3(5). The fluorescent signal was measured at λ_excitation_ = 620 nm and λ_emission_ = 670 with a microplate reader (Promega) every 5 min during 1 h. Furthermore, 0.1% Triton-X was used as a positive control. Data were normalized based on the fluorescent signal in the presence of 50 μL supplemented HEPES buffer and 150 μL diSC3(5)-bacterial suspension. The assay was carried out in triplicate.

### Statistical analyses.

For the biofilm experiments, the Dunnett’s T3 multiple comparison’s test was used to compare the means of the treated groups to the mean of the untreated control (GraphPad Prism 8).
