# Supplementary material for: In Vitro Evaluation of Antimicrobial Peptides from the Black Soldier Fly (Hermetia Illucens) against a Selection of Human Pathogens
Source: Microbiol Spectr. 2022 Jan 5;10(1):e01664-21. doi: 10.1128/spectrum.01664-21 (PMC8729770; doi:10.1128/spectrum.01664-21)
Supplement: SUPPLEMENTAL FILE 1 — Supplemental material. Download SPECTRUM01664-21_Supp_1_seq9.pdf, PDF file, 0.3 MB [file spectrum01664-21_supp_1_seq9.pdf]

**Table S1** Overview of the black soldier fly antimicrobial peptide library.

| Peptide | Sequence | Purity (%) | Modification | pI | Molar Mass (g/mol) |
|---|---|---|---|---|---|
| Alo1-like | | | | | |
| Hill-Alo1 | CINNGDGCQPDGRQGNCCSGYCHKEPGWVTGYCR | 90.94 | - | 6.67 | 3679.04 |
| Hill-Alo2 | CIANGNGCQPDGRQGNCCSGFCYKQRGWVAGYCRRR | 95.69 | - | 8.77 | 3985.5 |
| Diptericin fragment | | | | | |
| Hill-Dip1 | ASVPEPADLREAFETEDAIYIPISIEEANRLRLPR | 92.77 | - | 4.04 | 3982.41 |
| Hill-Dip2 | SAPKLEEESALSYIPDAAEAEIAEPASSHGRVRR | 96.53 | - | 3.37 | 3637.92 |
| Hill-Dip3 | DVEQVLETEDAYYVPVSDEEAEVLRLPR | 93.08 | - | 3.37 | 3264.5 |
| Hill-Dip4 | SIDDLTLSEDGEDHVEIITDDEVQRAKR | 93.25 | - | 3.7 | 3199.35 |
| Diptericin | | | | | |
| Hill-Dip5 | QLNIQGGGSPHSGFDLSVQGRAKIWESDNGRNTLYGTGQYGQH LGGPYGNSEPSFGGGLMFSHRF | Crude | - | 7.72 | 6928.42 |
| Hill-Dip6 | QIFAQGGGSPGKGYDIYAQGRAKLWESQNQRNSLHGTASYSQH LGGPYGNSRPNVGGGLTFTHRF | 90.00 | - | 10.28 | 6981.51 |
| Knottin-like | | | | | |
| Hill-Knot1 | IKCTASICTQICRILKYKCGYCASASRCVCLK | 91.56 | - | 8.79 | 3531.37 |
| Hill-Knot2 | IKCVPSQCNQICRVLGKKCGYCKNASTCVCL | 96.43 | C-terminal amidation | 8.81 | 3363.14 |
| Hill-Knot3 | RKCTASQCTRVCKKLGYKRGYCQSSTKCVC | 90.22 | - | 9.8 | 3390.06 |
| Defensin without SS[b] | | | | | |
| Hill-Def1 | VTCDLLEPFLGPAPCMIHCIVRFRKRTGYCNSQNVCVCR | 94.26 | C-terminal amidation | 8.41 | 4443.34 |
| Hill-Def2a | ATCDLLSPFKVGHAACAAHCIARGKRGGWCDKRAVCNCRK | 93.75 | - | 9.26 | 4275.04 |
| Hill-Def3 | ATCTNWNCRTQCIARGKRGGYCVERNICKCTS | 93.97 | - | 8.76 | 3597.16 |
| Hill-Def4 | ATCDLLSPFKVGHAACALHCIALGRRGGWCDGRAVCNCRR | 92.58 | - | 8.43 | 4259 |
| Hill-Def5 | AMCDLLSGLNMGRSVCAMRCILKGHRGGWCDDQGVCNCRV | 91.95 | - | 7.84 | 4330.15 |
| Hill-Def6 | LSCLFENQAVSAIACGASCITRKGKRGGWCSNGVCRCTPN | 90.56 | - | 8.42 | 4162.82 |
| Hill-Def7 | TTCDLISGTKIENIACAAHCIAMGHKGGYCNSNLICICR | 93.94 | - | 7.46 | 4099.82 |
| Hill-Def8 | QLPCDYLSGLGFGEDACNTDCIAKGHKSGFCTGLVCRCRTL | 92.20 | - | 6.66 | 4353.99 |
| Defensin with SS[b] | | | | | |
| Hill-Def2b | ATCDLLSPFKVGHAACAAHCIARGKRGGWCDKRAVCNCRK | 99.10 | SS-Bridges: 3-36; 16-30; 20-38 | 9.26 | 4275.04 |
| Linear peptide | | | | | |

| | | | | | |
|---|---|---|---|---|---|
| Hill-Lin1 | AIYHRSIRSPQFSGSSASANAQSSSGGRGGFGGSSSSSSANAET NSFGG | 92.77 | C-terminal amidation | 10.92 | 4712.8 |
| Hill-Lin2 | AIYHRSIRSPQFSGSSASANAQSSSGGRGGFGGSSSSSSANAET NSFGG | Crude | C-terminal amidation | 11.82 | 4712.8 |
| Cecropin | | | | | |
| Hill-Cec1 | SWWKKVFKPVEKLGQRVRDATIQGIGIAQQGANVLATVRGGPPQ | 96.40 | C-terminal amidation | 11.93 | 4785.52 |
| Hill-Cec2 | GWWKRVFKPVEKFGQRVRDAGVQGIAIAQQGANVLATARGGPP QQ | 90.49 | C-terminal amidation | 12.14 | 4873.54 |
| Hill-Cec3 | GWWKRVFKPVEKLGQRVRDAGIQGLEIAQQGANVLATARGGPP QQ | 90.85 | C-terminal amidation | 11.75 | 4911.59 |
| Hill-Cec4 | GWWKRVFKPVERLGQRVRDAGIQGLQIAQQGANVLATVRGGPP QQ | 90.12 | C-terminal amidation | 12.29 | 4966.67 |
| Hill-Cec5 | SWFKKVFKPVEKVGQRVRDAGIQGVAIAQQGANVLATARGGPPH | 91.89 | C-terminal amidation | 11.93 | 4669.36 |
| Hill-Cec6 | DQSKEPFKPMERPYQRVRDAKIQGITIAQNGPNVLAITRGGLEQ | 90.87 | C-terminal amidation | 10.54 | 4950.6 |
| Hill-Cec7 | GWWKKVFKPVEKLGQRVRDAGIQGIAIAQQGANVLATVRGGPPQ | 92.21 | C-terminal amidation | 11.93 | 4725.46 |
| Hill-Cec8 | GWWKRVFKPVEKFGQRVRDAGIQGIEIAQQGANVLATARGGPP QQ | 94.74 | C-terminal amidation | 11.75 | 4945.6 |
| Hill-Cec9 | GWWKRVFKPVEKLGQRVRDAGIQGLEIAQQGANVLATVRGGPP QQ | 91.69 | C-terminal amidation | 11.75 | 4939.64 |
| Hill-Cec10 | RWYKKIFKPVEKAVQRVRDGTLQALGVAQQAANVYATAQGAQQ QRHA | 92.13 | C-terminal amidation | 11.42 | 5279.98 |
| Hill-Cec11 | RSLWKKIFKPVEKLGQRVRDAGIQGIAIAQQGANVLATVRGGPPQ | 94.37 | C-terminal amidation | 12.16 | 4852.65 |
| Hill-Cec12 | RSLWKKLFKPVERAGQRIRDATIKGIVIAQQGANVLATIRGGPAIPP GQ | 91.34 | C-terminal amidation | 12.31 | 5277.19 |
| Hill-Cec13 | KSWWKKVFKPVERAGQRIRDASIKGLAVAQQGANVLSTIRGGPP VVAPPPQV | 91.28 | C-terminal amidation | 12.17 | 5589.51 |
| Stomoxyn-like | | | | | |
| Hill-Stom1 | FNNLPICVEGLAGDIGSILLGVESDIGALAGAIANLALIAGECAAQG EAGAAICA | Crude | C-terminal amidation | 3.27 | 5181.92 |
| Hill-Stom2 | TKAGPIVGNVLSIVGDAVAAIGIAIARAPAIVADVEYCATNVVATTV ATLSKFGAAVKKCA | 55.10 | C-terminal amidation | 9.4 | 5911.94 |

[a] The molecular weight (MW) and isoelectric point (pI) of the peptides were calculated using the online software at http://pepcalc.com/

[b] SS = disulfide bridges

**Table S2** IC50 values (concentrations causing 50% growth inhibition of the pathogen), MIC values (minimum inhibitory concentrations needed for visual absence of bacterial growth), and MBC values (minimum concentrations needed for bactericidal activity) obtained in two individual screenings: the first screening starting at 64µM, the second screening starting at 32 µM. Static = bacteriostatic activity.

| | | IC50 (µM) | | | | | | MIC (µM) | | | MBC (µM) | | |
|---|---|---|---|---|---|---|---|---|---|---|---|---|---|
| AMP | Repeat | MRC-5 SV2 | *S. aureus* ATCC 6538 | *E. coli* ATCC 8739 | *P. aeruginosa* ATCC 9027 | *C. albicans* B59630 | *A. fumigatus* B42928 | *S. aureus* ATCC 6538 | *E. coli* ATCC 8739 | *P. aeruginosa* ATCC 9027 | *S. aureus* ATCC 6538 | *E. coli* ATCC 8739 | *P. aeruginosa* ATCC 9027 |
| Hill-Alo1 | 1 | > 64.00 | > 64.00 | > 64.00 | > 64.00 | > 64.00 | > 64.00 | > 64.00 | > 64.00 | > 64.00 | - | - | - |
| | 2 | > 32.00 | > 32.00 | > 32.00 | > 32.00 | > 32.00 | > 32.00 | > 32.00 | > 32.00 | > 32.00 | - | - | - |
| Hill-Alo2 | 1 | > 64.00 | > 64.00 | > 64.00 | > 64.00 | > 64.00 | > 64.00 | > 64.00 | > 64.00 | > 64.00 | - | - | - |
| | 2 | > 32.00 | > 32.00 | > 32.00 | > 32.00 | > 32.00 | > 32.00 | > 32.00 | > 32.00 | > 32.00 | - | - | - |
| Hill-Dip1 | 1 | **32.00** | > 64.00 | > 64.00 | > 64.00 | > 64.00 | > 64.00 | > 64.00 | > 64.00 | > 64.00 | - | - | - |
| | 2 | > 32.00 | > 32.00 | > 32.00 | > 32.00 | > 32.00 | > 32.00 | > 32.00 | > 32.00 | > 32.00 | - | - | - |
| Hill-Dip2 | 1 | > 64.00 | > 64.00 | > 64.00 | > 64.00 | > 64.00 | > 64.00 | > 64.00 | > 64.00 | > 64.00 | - | - | - |
| | 2 | > 32.00 | > 32.00 | > 32.00 | > 32.00 | > 32.00 | > 32.00 | > 32.00 | > 32.00 | > 32.00 | - | - | - |
| Hill-Dip3 | 1 | > 64.00 | > 64.00 | > 64.00 | > 64.00 | > 64.00 | > 64.00 | > 64.00 | > 64.00 | > 64.00 | - | - | - |
| | 2 | > 32.00 | > 32.00 | > 32.00 | > 32.00 | > 32.00 | > 32.00 | > 32.00 | > 32.00 | > 32.00 | - | - | - |
| Hill-Dip4 | 1 | > 64.00 | > 64.00 | > 64.00 | > 64.00 | > 64.00 | > 64.00 | > 64.00 | > 64.00 | > 64.00 | - | - | - |
| | 2 | > 32.00 | > 32.00 | > 32.00 | > 32.00 | > 32.00 | > 32.00 | > 32.00 | > 32.00 | > 32.00 | - | - | - |
| Hill-Dip5 | 1 | > 64.00 | > 64.00 | > 64.00 | > 64.00 | > 64.00 | > 64.00 | > 32.00 | > 32.00 | > 32.00 | - | - | - |
| | 2 | > 32.00 | > 32.00 | > 32.00 | > 32.00 | > 32.00 | > 32.00 | > 64.00 | > 64.00 | > 64.00 | - | - | - |
| Hill-Dip6 | 1 | **4.54** | > 32.00 | **0.85** | > 32.00 | > 64.00 | > 64.00 | > 32.00 | **2.00** | > 3200 | - | static | - |
| | 2 | **7.73** | > 32.00 | **1.00** | > 32.00 | > 32.00 | > 32.00 | > 64.00 | **2.00** | > 64.00 | - | 8,00 | - |
| Hill-Knot1 | 1 | > 64.00 | > 64.00 | > 64.00 | > 64.00 | > 64.00 | > 64.00 | > 64.00 | > 64.00 | > 64.00 | - | - | - |
| | 2 | > 32.00 | > 32.00 | > 32.00 | > 32.00 | > 32.00 | > 32.00 | > 32.00 | > 32.00 | > 32.00 | - | - | - |
| Hill-Knot2 | 1 | **9.73** | > 64.00 | > 64.00 | > 64.00 | > 64.00 | > 64.00 | > 64.00 | > 64.00 | > 64.00 | - | - | - |
| | 2 | **3.76** | > 32.00 | > 32.00 | > 32.00 | > 32.00 | > 32.00 | > 32.00 | > 32.00 | > 32.00 | - | - | - |
| Hill-Knot3 | 1 | > 64.00 | > 64.00 | > 64.00 | **22.63** | > 64.00 | > 64.00 | > 64.00 | > 64.00 | > 64.00 | - | - | - |
| | 2 | > 32.00 | > 32.00 | > 32.00 | > 32.00 | > 32.00 | > 32.00 | > 32.00 | > 32.00 | > 32.00 | - | - | - |

| | | | | | | | | | | | | | |
|---|---|---|---|---|---|---|---|---|---|---|---|---|---|
| Hill-Def1 | 1 | > 64.00 | > 64.00 | > 64.00 | > 64.00 | > 64.00 | > 64.00 | > 64.00 | > 64.00 | > 64.00 | - | - | - |
| | 2 | > 32.00 | > 32.00 | > 32.00 | > 32.00 | > 32.00 | > 32.00 | > 32.00 | > 32.00 | > 32.00 | - | - | - |
| Hill-Def2a | 1 | > 64.00 | **< 0.25** | **35.33** | **32.86** | > 64.00 | > 64.00 | **0.25** | > 64.00 | > 64.00 | static | - | - |
| | 2 | > 32.00 | **0.19** | > 32.00 | > 32.00 | > 32.00 | > 32.00 | **2.00** | > 32.00 | > 32.00 | static | - | - |
| Hill-Def3 | 1 | > 64.00 | > 64.00 | > 64.00 | > 64.00 | > 64.00 | > 64.00 | > 64.00 | > 64.00 | > 64.00 | - | - | - |
| | 2 | > 32.00 | > 32.00 | > 32.00 | > 3.00 | > 32.00 | > 32.00 | > 32.00 | > 32.00 | > 32.00 | - | - | - |
| Hill-Def4 | 1 | > 64.00 | **0.50** | > 64.00 | **32.00** | > 64.00 | > 64.00 | **1.00** | > 64.00 | > 64.00 | static | - | - |
| | 2 | > 32.00 | **0.70** | > 32.00 | > 32.00 | > 32.00 | > 32.00 | **8.00** | > 32.00 | > 32.00 | static | - | - |
| Hill-Def5 | 1 | > 64.00 | > 64.00 | > 64.00 | > 64.00 | > 64.00 | > 64.00 | > 64.00 | > 64.00 | > 64.00 | - | - | - |
| | 2 | > 32.00 | > 32.00 | > 32.00 | > 32.00 | > 32.00 | > 32.00 | > 32.00 | > 32.00 | > 32.00 | - | - | - |
| Hill-Def6 | 1 | > 64.00 | > 64.00 | > 64.00 | > 64.00 | > 64.00 | > 64.00 | > 64.00 | > 64.00 | > 64.00 | - | - | - |
| | 2 | > 32.00 | > 32.00 | > 32.00 | > 32.00 | > 32.00 | > 32.00 | > 32.00 | > 32.00 | > 32.00 | - | - | - |
| Hill-Def7 | 1 | > 64.00 | > 64.00 | > 64.00 | > 64.00 | > 64.00 | > 64.00 | > 64.00 | > 64.00 | > 64.00 | - | - | - |
| | 2 | > 32.00 | > 32.00 | > 32.00 | > 32.00 | > 32.00 | > 32.00 | > 32.00 | > 32.00 | > 32.00 | - | - | - |
| Hill-Def8 | 1 | > 64.00 | > 64.00 | > 64.00 | > 64.00 | > 64.00 | > 64.00 | > 64.00 | > 64.00 | > 64.00 | - | - | - |
| | 2 | > 32.00 | > 32.00 | > 32.00 | > 32.00 | > 32.00 | > 32.00 | > 32.00 | > 32.00 | > 32.00 | - | - | - |
| Hill-Def2b | 1 | > 64.00 | **8.50** | > 64.00 | > 64.00 | > 64.00 | > 64.00 | **64.00** | > 32.00 | > 32.00 | static | - | - |
| | 2 | > 32.00 | **13.45** | > 32.00 | > 32.00 | > 32.00 | > 32.00 | > 32.00 | > 64.00 | > 64.00 | static | - | - |
| Hill-Lin1 | 1 | > 64.00 | > 64.00 | > 64.00 | > 64.00 | > 64.00 | > 64.00 | > 64.00 | > 64.00 | > 64.00 | - | - | - |
| | 1 | > 64.00 | > 64.00 | > 64.00 | > 64.00 | > 64.00 | > 64.00 | > 64.00 | > 64.00 | > 64.00 | - | - | - |
| Hill-Lin2 | 2 | > 32.00 | > 32.00 | > 32.00 | > 32.00 | > 32.00 | > 32.00 | > 32.00 | > 32.00 | > 32.00 | - | - | - |
| | 2 | > 32.00 | > 32.00 | > 32.00 | > 32.00 | > 32.00 | > 32.00 | > 32.00 | > 32.00 | > 32.00 | - | - | - |
| Hill-Cec1 | 1 | > 64.00 | > 64.00 | **< 0.25** | **< 0.25** | > 64.00 | > 64.00 | > 64.00 | **0.25** | **1.00** | - | 1.00 | 4.00 |
| | 2 | > 32.00 | > 32.00 | **0.21** | **0.20** | > 32.00 | > 32.00 | > 32.00 | **0.50** | **0.50** | - | 0.50 | 0.50 |
| Hill-Cec2 | 1 | > 64.00 | > 64.00 | **0.50** | **0.93** | > 64.00 | > 64.00 | > 64.00 | **1.00** | **4.00** | - | 1.00 | 16.00 |
| | 2 | > 32.00 | **4.36** | **0.31** | **0.76** | > 32.00 | > 32.00 | > 32.00 | **2.00** | **2.00** | - | 2.00 | 2.00 |
| Hill-Cec3 | 1 | > 64.00 | > 64.00 | **0.49** | **0.40** | > 64.00 | > 64.00 | > 64.00 | **1.00** | **4.00** | - | 1.00 | 16.00 |
| | 2 | > 32.00 | > 32.00 | **0.22** | **0.79** | > 32.00 | > 32.00 | > 32.00 | **0.50** | **2.00** | - | 32.00 | 2.00 |
| Hill-Cec4 | 1 | 37.37 | > 64.00 | **0.50** | **< 0.25** | > 64.00 | > 64.00 | > 64.00 | **1.00** | **1.00** | - | static | 16.00 |
| | 2 | > 32.00 | > 32.00 | **0.32** | **0.22** | > 32.00 | > 32.00 | > 32.00 | **2.00** | **2.00** | - | 2.00 | 2.00 |
| Hill-Cec5 | 1 | > 64.00 | > 64.00 | **0.50** | **2.61** | > 64.00 | > 64.00 | > 64.00 | **1.00** | **16.00** | - | static | static |

| | | | | | | | | | | | | | |
|---|---|---|---|---|---|---|---|---|---|---|---|---|---|
| | 2 | > 32.00 | > 32.00 | **0.23** | **3.60** | > 32.00 | > 32.00 | > 32.00 | **0.50** | **32.00** | - | 0.50 | 32.00 |
| Hill-Cec6 | 1 | > 64.00 | > 64.00 | > 64.00 | > 64.00 | > 64.00 | > 64.00 | > 64.00 | > 32.00 | > 32.00 | - | 1.00 | 16.00 |
| | 2 | > 32.00 | > 32.00 | > 32.00 | > 32.00 | > 32.00 | > 32.00 | > 32.00 | > 64.00 | > 64.00 | - | 2.00 | 2.00 |
| Hill-Cec7 | 1 | > 64.00 | > 64.00 | **0.38** | **0.40** | > 64.00 | > 64.00 | > 64.00 | **1.00** | **4.00** | - | - | - |
| | 2 | > 32.00 | > 32.00 | **0.32** | **0.78** | > 32.00 | > 32.00 | > 32.00 | **2.00** | **2.00** | - | - | - |
| Hill-Cec8 | 1 | > 64.00 | > 64.00 | **0.45** | **1.29** | > 64.00 | > 64.00 | > 64.00 | **1.00** | **4.00** | - | 1.00 | 64.00 |
| | 2 | > 32.00 | > 32.00 | **0.68** | **1.73** | > 32.00 | > 32.00 | > 32.00 | **2.00** | **8.00** | - | 8.00 | 8.00 |
| Hill-Cec9 | 1 | > 64.00 | > 64.00 | **0.43** | **0.38** | > 64.00 | > 64.00 | > 64.00 | **1.00** | **1.00** | - | 1.00 | static |
| | 2 | > 32.00 | > 32.00 | **0.21** | **0.88** | > 32.00 | > 32.00 | > 32.00 | **0.50** | **2.00** | - | 0.50 | 2.00 |
| Hill-Cec10 | 1 | > 64.00 | 32.74 | **< 0.25** | **< 0.25** | > 64.00 | > 64.00 | > 64.00 | **0.25** | **1.00** | - | 0.25 | 16.00 |
| | 2 | > 32.00 | > 32.00 | **0.22** | **0.22** | > 32.00 | > 32.00 | > 32.00 | **0.50** | **2.00** | - | 32.00 | 2.00 |
| Hill-Cec11 | 1 | > 64.00 | > 64.00 | **< 0.25** | **< 0.25** | > 64.00 | > 64.00 | > 64.00 | **1.00** | **1.00** | - | 1.00 | 4.00 |
| | 2 | > 32.00 | > 32.00 | **0.22** | **0.24** | > 32.00 | > 32.00 | > 32.00 | **0.50** | **2.00** | - | 32.00 | 8.00 |
| Hill-Cec12 | 1 | > 64.00 | > 64.00 | **< 0.25** | **< 0.25** | > 64.00 | > 64.00 | > 64.00 | **0.25** | **1.00** | - | 1.00 | static |
| | 2 | 19.45 | > 32.00 | **0.32** | **0.23** | > 32.00 | > 32.00 | > 32.00 | **2.00** | **2.00** | - | 8.00 | 8.00 |
| Hill-Cec13 | 1 | > 64.00 | > 64.00 | **< 0.25** | **< 0.25** | > 64.00 | > 64.00 | > 64.00 | **1.00** | **1.00** | - | 1.00 | 64.00 |
| | 2 | > 32.00 | > 32.00 | **0.23** | **0.25** | > 32.00 | > 32.00 | > 32.00 | **0.50** | **2.00** | - | 0.50 | 2.00 |
| Hill-Stom1 | 1 | **11.65** | **58.58** | > 64.00 | **16.00** | > 64.00 | > 64.00 | > 64.00 | > 32.00 | > 32.00 | - | 1.00 | 4.00 |
| | 2 | **24.42** | > 32.00 | > 32.00 | **17.67** | > 32.00 | > 32.00 | > 32.00 | > 64.00 | > 64.00 | - | 0.50 | 0.50 |
